# Investigating Wild Berries as a Dietary Approach to Reducing the Formation of Advanced Glycation Endproducts: Chemical Correlates of *In Vitro* Antiglycation Activity

**DOI:** 10.1007/s11130-014-0403-3

**Published:** 2014-01-22

**Authors:** Cory S. Harris, Alain Cuerrier, Erin Lamont, Pierre S. Haddad, John T. Arnason, Steffany A. L. Bennett, Timothy Johns

**Affiliations:** 1 School of Dietetics and Human Nutrition, McGill University, Montréal, QC Canada; 2 Department of Biology, University of Ottawa, 30 Marie Curie, Ottawa, Canada K1N 6N5; 3 Department of Biochemistry, Microbiology, and Immunology, University of Ottawa, 30 Marie Curie, Ottawa, Canada K1N 6N5; 4 Département de sciences biologiques, Université de Montréal, Montréal, QC Canada; 5 Département de pharmacologie, Université de Montréal, Montréal, QC Canada

**Keywords:** Fruits, Aging, Chronic disease, Oxidative stress, Radical scavenging, Polyphenolics, Anthocyanins

## Abstract

Evidence supports the health promoting benefits of berries, particularly with regard to the prevention and management of chronic diseases such cardio- and cerebrovascular disease, diabetes and Alzheimer’s disease. Two related pathophysiological features common to many of these conditions are oxidative stress and the accumulation of advanced glycation endproducts (AGEs). Whereas antioxidant properties are well-established in several species of berries and are believed central to their protective mechanisms, few studies have investigated the effects of berries on AGE formation. Here, employing a series of complementary *in vitro* assays, we evaluated a collection of wild berry extracts for 1) inhibitory effects on fluorescent-AGE and Nε- (carboxymethyl)lysine-albumin adduct formation, 2) radical scavenging activity and 3) total phenolic and anthocyanin content. All samples reduced AGE formation in a concentration-dependent manner that correlated positively with each extract’s total phenolic content and, to a lesser degree, total anthocyanin content. Inhibition of AGE formation was similarly related to radical scavenging activities. Adding antiglycation activity to the list of established functional properties ascribed to berries and their phenolic metabolites, our data provide further insight into the active compounds and protective mechanisms through which berry consumption may aid in the prevention and treatment of chronic diseases associated with AGE accumulation and toxicity. As widely available, safe and nutritious foods, berries represent a promising dietary intervention worthy of further investigation.

## Introduction

The potential benefits of fruit consumption to human health have long been recognized. Recently, several berries^1^ have emerged as excellent sources of phytonutrients with putative protective effects toward age-related chronic diseases [1]. Promising evidence in human, animal and cellular studies specifically links berry consumption to lower risk of cardiovascular disease, diabetes and associated complications, cancer, and neurodegenerative diseases [2–5]. In addition to providing a valuable source of vitamins, minerals, and dietary fiber, berries contain a diversity of secondary metabolites, most notably phenolics, which show complex biological activity [6].

Berry phenolics range in structural complexity, from simple phenolic acids to polyphenolic tannins. Although present in many fruits, certain types of berries are remarkably rich in phenolic metabolites – particularly anthocyanins [7]. Responsible for the red, blue and purple colours characteristic of many berries, anthocyanins are extensively studied and often listed as medicinal ingredients of berry-based health products. Beyond antioxidant properties, berry phenolics (including anthocyanins) display anti-inflammatory, cardioprotective, neuroprotective and hypoglycemic activities through modulation of multiple signaling pathways implicated in the development of chronic and degenerative diseases [8–10]. Many phenolics also inhibit the formation of advanced glycation endproducts (AGEs) [11].

The accumulation and ensuing physiological consequences of AGEs often appear in patients with diabetes, cardiovascular, renal and neurodegenerative diseases as well as some types of cancer [12,13]. Produced through non-enzymatic reactions between reducing sugars and proteins, nucleic acids, or lipids, AGEs induce oxidative stress and molecular cross linkages that cause cellular and tissue damage by impairing protein function and clearance [14]. Several AGEs, such as Nε-(carboxymethyl)lysine (CML), activate the cell surface AGE receptor (RAGE) to initiate NF-κB and associated proinflammatory signaling pathways [14,15]. Whereas AGEs are produced endogenously under normal physiological conditions, they accumulate with age, developing more readily in hyperglycaemic and oxidative conditions. Despite the known antioxidant activity of berries and antiglycation activity of related phenolics, only few individual species have been assessed for effects on AGE formation [16,17]. In these studies, phenolic metabolites are consistently identified as active constituents [16].

Here, using complementary *in vitro* assays, we evaluate 12 species of wild berries collected from northern Québec, Canada, for their effects on AGE formation. Predicting that antiglycation activity will correlate positively with both phenolic content and anti-oxidant activity, we also assess each extract for total phenolic content, anthocyanin content and radical scavenging capacity.

## Materials and Methods

### Reagents

Chemicals and solvents were purchased from Fisher Scientific (Ottawa, ON, Canada) unless otherwise specified.

### Plant Materials and Extraction

Ripe berries and voucher specimens were collected from various locations in Québec, Canada. Upon maturity (August–September), samples were harvested, cleaned, and frozen on site prior to transportation to the University of Ottawa for extraction. Voucher specimens were deposited in the University of Ottawa Herbarium in Ottawa, Canada, with identities confirmed by Dr. Alain Cuerrier. Twenty grams of frozen berries from each collection were lyophilized to dryness, crushed with a mortar and pestle then extracted three times with 25 mL of 80 % ethanol per g dry material. Filtered extracts were pooled prior to drying using a Speedvac System AE52010 (Savant, Halbrook, NY) at 43 °C followed by lyophilization. Dried extracts were stored at 4 °C in darkness.

### AGE Formation Assay

Effects on AGE formation were determined as previously described [16]. Briefly, 100 mM sodium phosphate monobasic monohydrate buffer (pH 7.4) with 100 mM glucose/100 mM fructose (BDH Chemicals Ltd., Toronto, ON) and bovine serum albumin (BSA) (Sigma Aldrich, St. Louis, MO, USA) (1 mg/mL) were incubated with vehicle (negative control), berry extract (treatment), or quercetin (positive control) in darkness for seven days at 37 °C on a mechanical shaker (Series 25 Incubator Shaker, New Brunswick Scientific Co. Inc., Edison, NJ). Each plate included positive and negative controls as well as replicates without BSA to correct for analyte autofluorescence and without sugar to: 1) control for non-AGE-derived fluorescence of BSA, and 2) serve as a negative control for immunochemical assessments. Extracts were tested at concentrations of 12.5 – 400 μg/ml, in quadruplicate, on at least three separate occasions.

### Detection and Quantitation of Fluorescent AGEs and CML-BSA Adducts

Fluorescent AGEs were measured at excitation and emission wavelengths of 355 and 460 nm, respectively, using a SpectraMax M5 microplate reader (Molecular Devices, Sunnyvale, California). After measurement, plates were sealed with parafilm and stored at −20 °C for immunochemical analyses. Percent inhibition was calculated with corrected fluorescence values (F): *%* inhibition = [(F_negative_ − F_experimental_)/(F_negative_)] × 100 *%* [18]. IC_50_ concentrations, defined as the amount of extract (μg/ml) or positive control (μM) required to reduce AGE formation by 50 % relative to the negative control, were determined by logarithmic regression analyses (*n* = 3–4).

To detect and quantitate CML-BSA adduct formation [18], samples from control and extract-treated wells were first concentrated using Ultracel YM-10 membrane centrifugal filters (Millipore, Billerica, MA, USA). BSA concentrations were established by Bio-Rad DC Protein assay Kit (Bio-Rad Laboratories Ltd., Mississauga, ON, CAN). After protein separation by SDS-PAGE under reducing conditions, Western blot analyses were performed using mouse monoclonal antibodies targeted against CML-BSA adducts (1:1000, Clone 318003, R & D Systems, Minneapolis, MN, USA) and goat anti-mouse IgG polyclonal antibodies conjugated with horseradish peroxidase (1:1000, R & D Systems). Blots were treated with SuperSignal West Pico (MJS BioLynx Inc) to visualize adducts.

To qualitatively evaluate concentration-dependent effects, protein samples treated with 25, 50, and 100 μg/mL of the same extract were loaded on a single gel. Quantitative comparisons were conducted on blots of samples treated with different extracts administered at 50 μg/mL. Gels stained with Coomassie Blue and photographed following electrophoresis served as loading controls. Immunoreactive and Coumassie stained bands were quantified by image densitometry (ImageJ software v.1.38X, National Institutes of Health, Bethesda, MD). After correcting for protein loading, we calculated percent inhibition relative to vehicle control (*n* = 4–6).

### Measurement of Total Phenolic and Total Anthocyanin Content

Total phenolic content (TPH) was determined via a modified Folin-Ciocalteau method [19]. Freshly diluted Folin-Ciocalteau reagent (BDH, Toronto, ON) was combined with extract re-solubilized in 80 % ethanol and, after 5 min of equilibration, 7.5 % anhydrous NaHCO_3_ solution. After 2 h incubation at 23 °C, absorbance was measured at 725 nm and TPH values were calculated and expressed as quercetin (Sigma, Oakville, ON) equivalents. Experiments were conducted in quadruplicate on at least three separate occasions (*n* = 3–4).

Total monomeric anthocyanin content (TMAC) was measured following the acid-differential method [20]. Duplicate samples of each berry extract were dissolved in 0.025 M potassium chloride buffer, one adjusted to pH 1.0 and the other to pH 4.5 using 0.4 M sodium acetate buffer. After 15 min of incubation at RT, absorbance values at 496 and 700 nm were obtained with a Beckman DU-640 Spectrophotometer (Fullerton, CA). TMAC was calculated and expressed as cyanidin-3-glucoside (Fluka, Lyon, France) equivalents using corresponding molar absorptivity (26,900 L/cm·mol) and molecular weight (449.2 g/mol). Experiments were conducted in quadruplicate on three or more occasions (*n* = 3–4).

### Radical Scavenging Assays

Oxygen radical absorbance capacity (ORAC) was measured as previously described [18] in 75 mM phosphate buffer solution (PBS), pH 7.0. Freshly prepared vehicle (50 % MeOH/50 % PBS), Trolox (0.006-12.5 μM), ascorbic acid (0.006-12.5 μM), quercetin (0.002-3.13 μM) or berry extract (0.006-12.5 μg/mL) were loaded in black 96-well microplates, mixed with 0.08 μM fluorescein and allowed to incubate at 37 °C for 10 min before adding 150 mM AAPH. Fluorescence was monitored with a Synergy HT Multi-detection microplate reader (BioTek Instruments, Inc., Winooski, VT) every three min for 90 min at excitation and emission wavelengths of 485 nm and 530 nm, respectively. ORAC values were calculated as μM Trolox equivalents per mg dried extract as determined from at least four separate experiments (*n* = 4–5).

A modified 1,1-diphenyl-2-picrylhydrazyl (DPPH) radical assay [18] provided a second measure of free radical scavenging activity. Ascorbic acid (0–60 μM), quercetin (0–60 μM), or extract (0–1,000 mg/mL) dissolved in methanol was combined with 100 μM DPPH and allowed to stand at RT for 10 min before we measured absorbance at 517 nm using a Beckman DU 640 spectrophotometer. EC_50_, the concentration of extract (μg/mL) or positive control (μM) required to scavenge 50 % of radicals, was determined by linear regression analyses (*n* = 4) with relative scavenging capacity expressed as % ascorbic acid equivalents (*%* AAE = [(EC_50_)_ascorbic acid_/(EC_50_)_treatment_] × 100*%*.

### Statistical Analyses

Results are expressed as means ± SEM. Pearson’s correlation analyses were used to investigate relationships between TPH, TMAC, antiglycation and radical scavenging activities, using Log-transformed data, as required. Correlation coefficients, coefficients of determination (r^2^) and P-value estimates were calculated using S-Plus 8.0 statistical software (Insightful Corp., Seattle, WA).

## Results

Fourteen samples representing 12 species are detailed in Table 1. Three samples of *V. uliginosum* were included to gauge potential intra-species variation. Water content was over 75 % for all berries except *J. communis*, a modified cone more frequently used for medicine than food. Extraction yields varied from 36 to 79 % of dry weight (Table 1).

**Table 1:** List of sampled berry species with collection details and extraction data

Species	Family	Common name	Source ^a^	Colour	% water ^b^	% yield ^c^
*Arctous alpina* L.	Ericaceae	Black bearberry	1	Dark purple	79.5	35.6
*Cornus canadensis* L.	Cornaceae	Bunchberry	2	Red	89	48.8
*Empetrum nigrum* L.	Ericaceae	Crowberry	2	Dark purple	86	42.1
*Juniperus communis* L.	Pinaceae	Common juniper	2	Blue	68	47.2
*Ribes glandulosum* Grauer	Grossulariaceae	Swamp currant	2	Red	78.5	64.8
*Ribes triste* Pall.	Grossulariaceae	Smooth currant	2	Red	76	43.5
*Rubus chamaemorus* L.	Rosaceae	Cloudberry	1	Orange	84	62.7
*Sorbus decora* (Sarg.) CK Schneid.	Rosaceae	Showy mountain ash	2	Orange-red	81	72.8
*Vaccinium angustifolium* Ait.	Ericaceae	Lowbush blueberry	3	Blue	85	74.1
*Vaccinium oxycoccus* L.	Ericaceae	Bog cranberry	4	Red	77.5	44.0
*Vaccinium uliginosum* L. (A)	Ericaceae	Bog bilberry	1	Blue	84.5	73.3
*V. uliginosum* L. (B)	Ericaceae		2	Blue	85.5	76.0
*V. uliginosum* L. (C)	Ericaceae		2	Dark purple	87	78.1
*Vaccinium vitis-idaea* L.	Ericaceae	Lingonberry	4	Crimson	85	79.2

^a^Berry samples were collected in August and September near: 1) Kuujuac, Québec, Canada; 2) Great Whale River, Québec, Canada; 3) La Vérendrye Wildlife Reserve, Québec, Canada; 4) Chibougamau, Québec, Canada

^b^Percent water of berry samples is expressed as [1 − (g dry wt/g fresh wt)] × 100*%*

^c^Percent yield is expressed as (g extract/g dry wt) × 100*%*

### Inhibition of Non-Enzymatic Formation of AGEs

**Fig. 1:**
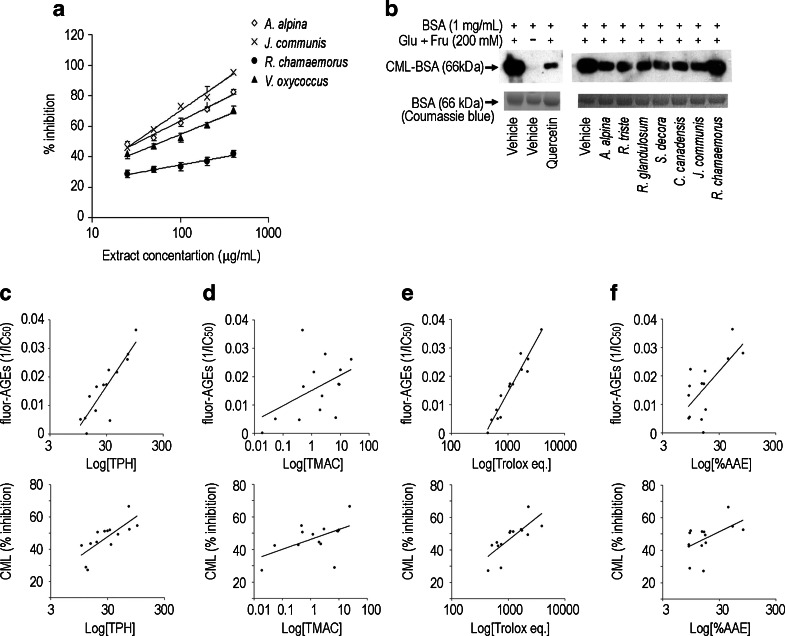
Inhibition of AGE formation by representative berry extracts and correlations with phenolic and anthocyanin content as well as radical scavenging activity. **a** Concentration-dependent inhibition of fluorescent AGE formation by various extracts (mean ± SEM, *n* = 3–4). Corresponding IC_50_ values are listed in Table 2. **b** Western blot using CML-specific antibodies on protein samples incubated with glucose/fructose, vehicle, and quercetin (positive control, 16 μM), or 50 μg of berry extract. BSA incubated without sugar was used as a negative control for the CML-BSA antibody. Each SDS-PAGE separation was performed in duplicate; the first was used for Western blotting (*upper panels*) and the second to control for protein loading (Coomassie Blue staining – *lower panels*). **c**-**f** Pearson’s correlation analyses of fluorescent AGE (fluor-AGE) and carboxymethyl lysine (CML) inhibition relative to total phenolic content (TPH, expressed as quercetin equivalents), total monomeric anthocyanin content (TMAC, expressed as cyanidin-3-glucoside equivalents), oxygen radical absorbance capacity (ORAC, expressed as Trolox equivalents), and 1,1-diphenyl-2-picrylhydrazyl (DPPH) radical scavenging activity expressed as percent ascorbic acid equivalents (% AAE). [(**c**) fluor-AGE vs. TPH: *p* < 0.001; *r* ^2^ = 0.760; CML vs TPH: *p* = 0.001; *r* ^2^ = 0.594; (**d**) fluor-AGE vs. TMAC: *p* = 0.097; *r* ^2^ = 0.213; CML vs TMAC: *p* = 0.047; *r* ^2^ = 0.291; (**e**) fluor-AGE vs. Trolox eq: *p* < 0.001; *r* ^2^ = 0.928; CML vs Trolox eq: *p* = 0.002; *r* ^2^ = 0.578; (**f**) fluorAGE vs. %AAE: *p* < 0.008; *r* ^2^ = 0.452; CML vs %AAE: *p* = 0.059; *r* ^2^ = 0.266]

Fluorometric analyses of AGEs revealed concentration-dependent inhibition by all extracts but with varying potencies (Fig. 1a). Whereas most IC_50_ values were above 50 μg/mL, those of five extracts (*J. communis*, *A. alpina*, *E. nigrum*, *V. vitis-idaea,* and *R. glandulosum*) were approximately 100-fold lower than the weakest inhibitor, *R. chamaemorus* (Table 2). Quercetin, a commonly occurring flavonoid with established AGE inhibiting activity, exhibited an IC_50_ of 6.1 μM while ascorbic acid elicited no effect at the tested concentrations (0.2–200 μM). Immunochemically detected CML-BSA adducts in vehicle-control samples (with BSA + glucose/fructose) established the uninhibited level of glycation (100 %), vehicle with BSA alone yielded no adducts, and quercetin (16 μM) reduced adduction by over 80 % (Fig. 1b). All extracts inhibited CML-BSA adduct formation in a concentration-dependent manner (data not shown) with relative activities (at 50 μg/mL) illustrated in Fig. 1b. Percent inhibition relative to the vehicle-controls ranged from 37 % for *R. chamaemorus* to 77 % for *E. nigrum* (Table 2) and correlated highly with IC_50_ values obtained in the fluorescence assay (*p* < 0.001; *r* ^2^ = 0.651).

**Table 2:** Antiglycation activities, total phenolic and total anthocyanin contents, and radical scavenging activities of berry extracts

Treatment	AGE inhibition		Phenolic & anthocyanin content		Anti-oxidant activity	
	Fluorescent AGE - IC_50_^a^	CML-BSA % inhibition ^b^	TPH ^c^ (mg/g)	TMAC ^d^ (mg/g)	ORAC ^e^ Trolox eq.	DPPH ^f^ % AAE
Quercetin	6.1 ± 1.8	81.4 ± 5.6	–	–	91845 ± 9437	205 ± 13.3
Ascorbic acid	No effect	–	–	–	3669 ± 274	100 ± 4.7
*A. alpine*	35.6 ± 9.5	62.6 ± 82.0	71.9 ± 3.1	2.91 ± 0.20	1660 ± 136	75.4 ± 8.9
*C. canadensis*	120.3 ± 6.9	54.6 ± 4.0	19.2 ± 4.2	1.93 ± 0.03	624 ± 38	19.4 ± 2.2
*E. nigrum*	38.2 ± 2.5	76.7 ± 7.2	70.0 ± 5.7	23.27 ± 1.37	2247 ± 225	40.2 ± 6.9
*J. communis*	27.4 ± 2.6	64.9 ± 4.4	99.2 ± 4.7	0.47 ± 0.07	3876 ± 142	48.6 ± 2.3
*R. glandulosum*	46.0 ± 4.2	59.4 ± 5.0	45.4 ± 6.5	1.17 ± 0.12	2182 ± 109	16.8 ± 2.5
*R. triste*	208.6 ± 34.4	53.0 ± 4.4	34.2 ± 7.7	0.36 ± 0.15	510 ± 34	22.8 ± 5.7
*R. chamaemorus*	4009 ± 1753	37.4 ± 14.8	13.2 ± 2.3	0.02 ± 0.01	438 ± 57	19.4 ± 1.9
*S. decora*	192.7 ± 37.1	52.6 ± 1.5	10.3 ± 1.7	0.05 ± 0.01	657 ± 88	10.8 ± 1.7
*V. angustifolium*	177.8 ± 22.4	39.0 ± 3.7	12.4 ± 2.1	6.81 ± 0.50	732 ± 38	9.5 ± 1.6
*V. oxycoccus*	60.2 ± 1.5	60.9 ± 4.1	19.8 ± 3.1	0.50 ± 0.13	1049 ± 260	7.7 ± 0.7
*V. uliginosum (A)*	75.5 ± 14.7	53.6 ± 3.9	14.9 ± 1.9	2.27 ± 0.03	747 ± 29	8.0 ± 0.7
*V. uliginosum (B)*	57.5 ± 14.6	61.5 ± 7.1	29.8 ± 5.1	8.55 ± 0.32	1075 ± 132	13.8 ± 2.2
*V. uliginosum (C)*	57.8 ± 8.2	61.4 ± 4.4	26.3 ± 6.4	9.07 ± 0.35	1216 ± 112	15.3 ± 3.2
*V. vitis-idaea*	44.5 ± 12.4	62.1 ± 4.7	32.6 ± 2.2	9.84 ± 0.37	1725 ± 66	8.1 ± 0.8

^a^IC_50_ concentrations ± SEM calculated as the extract (μg/mL) or control (μM) concentration required to reduce fluorescent AGE formation by 50 % as determined by regression analysis (*n* = 3–4)

^b^% inhibition ± SEM of Nε-(carboxymethyl)lysine-BSA (CML-BSA) adduct formation as determined by densitometry of Western blot analyses of purified protein following treatment with sugar, vehicle, 16 μM quercetin or 50 μg extract/mL (*n* = 4–5)

^c^Total phenolic content (TPH) as determined by the Folin-Ciocalteu method, expressed as mg quercetin equivalents/g extract ± SEM (*n* = 3–4)

^d^Total monomeric anthocyanin content (TMAC) as determined by the acid differential method, expressed as mg cyaniding-3-glucoside equivalents/g extract ± SEM (*n* = 3–4)

^e^Oxygen radical absorbance capacity (ORAC) is expressed in μM Trolox equivalents ± SEM at a concentration of 1 mg/mL for extracts and 1 mM for quercetin and ascorbic acid (*n* = 4–5)

^f^Percent ascorbic acid equivalents (% AAE) as determined by the 1,1-diphenyl-2-picrylhydrazyl (DPPH) radical scavenging assay (*n* = 4–5)

### Total Phenolic and Anthocyanin Content

TPH ranged from 10 to 100 mg quercein equivalents per g extract, highest in *J. communis*, *A. alpina*, and *E. nigrum* extracts and lowest in *S. decora*, *V. angustifolium* and *R. chamaemorus,* extracts (Table 2). TMAC, a subclass of phenolics, was expectedly lower than TPH in all extracts and was highest in Ericaceous berries (with the exception of *V. oxycoccus*). No correlation, however, was observed between the two measures.

### Radical Scavenging Activity

Extracts of *J. communis*, *E. nigrum*, and *R. glandulosum* generated the strongest scavenging effects with ORAC values over 2,000 μM Trolox equivalents (TE). In contrast, the TE of six extracts (*C. canadensis, R. triste, R. chamaemorus*, *S. decora, V. angustifolium* and *V. oxycoccus*) was below 700 μM (Table 2). Whereas the activity of ascorbic acid and the most potent extract (*J. communis*) were similar, that of quercetin was 20-times greater. In the DPPH assay, a similar 10-fold difference in activity was observed between the strongest and weakest radical scavenging extracts. Quercetin (EC_50_ = 12.3 ± 0.8 μM, 205 % AAE) was twice as effective as ascorbic acid (EC_50_ = 25.4 ± 1.2 μM) with extracts of *A. alpina* (78 % AAE), *J. communis* (51 % AAE) and *E. nigrum* (43 % AAE) demonstrating the most potent activities among berry samples (Table 2). Extracts of *S. decora* and the four species of *Vaccinium* demonstrated much weaker activity (<15 % AAE). ORAC and DPPH results were significantly correlated (*p =* 0.028; *r* ^*2*^ = 0.342).

### Correlations Between Biochemical Activities and Phenolic or Anthocyanin Content

Pearson’s correlation analyses detected several significant, positive relationships among the investigated variables (Fig. 1). TPH was highly correlated with all measures of antiglycation and radical scavenging activity but correlations between TMAC and AGE inhibition were less pronounced, only significant in relation to CML-BSA adducts (Fig. 1c-d). Inhibition of AGE formation, particularly fluorescent AGEs, was strongly correlated with ORAC scores (Fig. 1e) while DPPH radical scavenging correlated less robustly with fluorescent AGE formation and insignificantly with CML-BSA adduction (Fig. 1f).

## Discussion

Within this collection of Canadian berry extracts, antiglycation activity correlates strongly with radical scavenging activity and total phenolic content as well as weakly with total anthocyanin content. Given that the AGE-inhibiting effects of other small fruits – muscadine grape [17], lingonberry [16] and acerola [21] – have been attributed to phenolic metabolites including but not limited to anthocyanins, this finding is not surprising. As such, while anthocyanins are effective AGE inhibitors contributing to activity, TPH appears more pertinent than TMAC to antiglycation (and radical scavenging) activity, in general.

Inhibition of AGE formation correlated more strongly with ORAC data than with those for DPPH scavenging, likely reflecting the greater involvement of peroxyl- over DPPH-like radicals in glycation reactions and/or inhibitory mechanisms. The relative activities of quercetin and ascorbic acid – both commonly found in berries – provide additional insight since 1) quercetin was 20 times more effective than ascorbic acid in the ORAC assay but only twice so in the DPPH assay, and 2) quercetin potently inhibits AGE formation while ascorbic acid had no effect in our system (and enhances glycaton in others [22]). Apparently, not all antioxidants prevent glycation. Moreover, antiglycation activity is determined by more than antioxidant properties. Indeed, studies on individual plant phenolics report distinct structural determinants of antioxidant and antiglycation activity [11,23].

Of the three samples of *V. uliginosum*, the two James Bay collections (B & C) yielded nearly identical results in all assays. Sample A, collected in the far north, however, contained fewer phenolics and anthocyanins and displayed correspondingly weaker activities, highlighting the impact of intra-species variation. Because berry composition depends on genetic, developmental, seasonal, ecological and postharvest factors, the precise activities and rankings reported here are specific to the collected samples. However, as similar correlations between phenolics, radical scavenging, and AGE inhibition are found among medicinal plants of Canada [18] and Thailand [24], the identified relationships likely extend to other collections of berries and phenolic-containing plant foods.

Whereas the extract concentrations required to significantly reduce AGE formation exceeded 10 μg/mL, often by an order of magnitude, individual phenolics are active at concentrations below 1 μg/mL [18]. Despite low bioavailability resulting from poor absorption and rapid metabolic breakdown, plasma levels of food-derived phenolics can reach low μM concentrations [25]. Consumption of berries increases circulating levels of intact phenolics as well as their metabolites [26], some of which retain their antiglycation activity [27]. Increasing consumption of berries, particularly phenolic-rich species such as *E. nigrum* and *A. alpina*, may thus aid in the prevention and management of AGE-related conditions like diabetes and associated complications. Given that this is the first study to explore antiglycation relationships within a collection of plant-based foods, our results set the stage for future antiglycation research of phenolics and their human and microbial metabolites in cells, animals, or humans.

In northern Québec, access to fresh fruits and vegetables is limited and expensive when available. Whereas other wild fruits are scarce, berries are plentiful and contribute valuably to the traditional diet of the region’s indigenous communities, which, like many others around the world, suffer increased risk of diabetes and diabetic complications [28,29]. Representing a culturally relevant and locally accessible source of needed nutrients and plant phenolics, berries warrant continued investigation as dietary means of improving aboriginal health.

## Footnotes

1 In this paper, the term ‘berry’ is used in reference to small, soft fruits and includes fruits such as raspberry and strawberry that, botanically, are not true berries.
